# The threshold elemental ratio of carbon and phosphorus of *Daphnia magna* and its connection to animal growth

**DOI:** 10.1038/s41598-018-27758-7

**Published:** 2018-06-26

**Authors:** Hamza K. Khattak, Clay Prater, Nicole D. Wagner, Paul C. Frost

**Affiliations:** 1 Department of Biology, Trent University, Peterborough, Ontario K9L 0G2 Canada; 2 Environmental and Life Science Graduate Program, Trent University, Peterborough, Ontario K9L 0G2 Canada

## Abstract

The growth of animal consumers is affected by the balance of elements in their diet with the transition between limitation by one element to another known as the threshold elemental ratio (TER). Precise estimates of TERs with known levels of uncertainty have yet to be generated for most zooplankton consumers. We determined the TER for carbon (C) and phosphorus (P) in for a common lake zooplankter, *Daphnia magna*, using experimental measurements and theoretical considerations. *Daphnia* growth responses to food C:P ratios across a relatively narrow range (80–350) generated an empirical estimate of TER_C:P_ of 155 ± 14. While this TER matched our modelled estimate of TER_C:P_ (155 ± 16), it was lower than previous estimates of this dietary transition point. No threshold was found when we examined daphnid body C:N or C:P ratios in response to changing food C:P ratios, which indicates P-limitation at even lower food C:P ratios. Our results provide strong evidence that *D. magna* is likely to experience acute P-limitation when food C:P ratios exceed even relatively low ratios (~155). Our model further demonstrated that while physiological adjustments may reduce the likelihood of P-limitation or reduce its intensity, these changes in animal material processing would be accompanied by reduced maximum growth rates.

## Introduction

Nutrition is a central determinant of organismal performance due to its effects on life-history traits such as growth, reproduction, and survival^1,2^. Given the potential ecological consequences of inadequate nutrition on organisms and their role in ecosystems^3^, there is a continuing need to determine if, and when, animal consumers experience nutritional deficiency in their food sources^2^. One well-developed approach to assessing the frequency or intensity of nutrient limitation acting on consumers has been to calculate threshold elemental ratios (TER)^4,5^. The TER is the nutritional mixture where animals grow fastest and is characterized by high dietary and environmental supplies of all needed nutrients. It follows that when elemental ratios in food are above or below this ‘threshold’ ratio^6^, animal growth will be reduced. TERs are thus roughly equivalent to ideal or optimal nutrient ratios for primary producers^7^ and the ‘stoichiometric knife-edge’ for consumers^8,9^ as they delineate food conditions under which the organism is limited by one element or another.

Modelled estimates of TERs produced for aquatic consumers vary substantially within and among taxa^10^. Within taxa, this variability in TER estimates creates uncertainty about the nutritional requirements for maximal growth. For example, *Daphnia*, a well-studied zooplankter, is widely recognized to be sensitive to low food phosphorus (P) content^11–13^. Despite this, the TER where animals switch from carbon (C) to P limitation remains uncertain with modelled TER_C:P_ estimates ranging from 100 to nearly 400 (Table 1). This wide range of TER_C:P_ estimates partly reflects differences in assumptions regarding nutrient acquisition and in parameter values that represent the maximal efficiency of C and P assimilation. Generally, models that use low C assimilation efficiencies elevate TER_C:P_ and predict a lower likelihood of P-limitation in animal consumers^1,10^. Such uncertainty in TER_C:P_ and the food conditions under which animals experience dietary P-limitation has implications for understanding of zooplankton nutrition in lakes. For example, TER_C:P_ estimates on the low end of the range (~150 or lower) lead to predictions of more frequent and severe P-limitation of *Daphnia* whereas higher TER_C:P_ estimates (~250 and above) indicate P-limitation of daphnids should be uncommon and, when present, to be quite mild (e.g.,^14^).

**Table 1:** Estimates of *Daphnia* TER_C:P_ from previous modelling studies.

Source	TER_C:P_	Species	Notes
Anderson and Hessen^6^	200	*Daphnia* sp.	Expansion of Sterner^4^
Brett *et al*.^14^	300	*Daphnia* sp.	Modified Urabe and Watanabe^20^; Based on relatively low A_c_
Frost *et al*.^10^	100	*D. magna*	Relatively low R_c_, high I_c_ and A_c_ using simplified Sterner^4^
Frost *et al*.^28^	151	*D. magna*	Three element version of Sterner^4^
Sterner^4^	171	*D. magna*	Modified Urabe and Watanabe^20^ to include ingestion and respiration
Shimizu and Urabe^26^	170	*Daphnia* sp.	Following Anderson and Hessen^6^
Urabe and Watanabe^30^	385	*D. galeata*	Assumed very low GGE_c_ but not explicit with ingestion and respiration.

TER estimates presented assume non-limiting food concentrations.

Uncertainty about the frequency and intensity of dietary P-limitation may also partly reflect difficulties in empirically assessing animal growth responses to subtle changes in food quality. Growth responses to marginally poor nutrition may be unapparent due to animal acclimation, which allows for continued growth even if acute dietary limitation is present^15^. Physiological acclimation may reduce effect sizes of poor food quality despite a proximate nutritional deficiency^16^. In addition, precise manipulation of food quality across a narrow gradient can be technically difficult to achieve and may produce relatively small effects relative to residual experimental error. Past assessments of stoichiometric food quality and its effects generally focused on broad nutritional gradients and severe forms of nutritional limitation to avoid these experimental limitations. However, the use of a few but widely spaced food C:P ratios in growth response experiments would appear less capable of generating precise estimates of food C:P ratio above which P-limitation occurs in *Daphnia* or other invertebrate consumers.

Given the myriad of stoichiometric dynamics that emerge from nutrient-stressed consumers^3,17,18^, there is an obvious need to have a deeper understanding of the conditions that lead to nutritional stress. For example, incorporation of elemental limitation into predator-prey models has largely assumed linear relationships between food quality and animal life-history traits and distinct transition points between different types of elemental limitation^4,18^. These sharp transitions may be ‘modulated’ if consumers alter physiological processes, including ingestion and digestion, in response to poor stoichiometric food quality (e.g.,^19^). While ‘stoichiometric modulation’ has been proposed to decouple growth from food C:nutrient ratios^16^, its consequences on an animal’s likelihood of nutrient limitation and its growth rate remain unclear. One approach to studying the implications of consumer physiological performance is to explicitly connect models of TER and animal growth.

Here we estimated the TER_C:P_ of *Daphnia magna* using a combination of growth experiments and individual mass balance modelling. With our experiments, we grew animals on algal food across a relatively narrow food C:P ratio range (~100 to ~300). We used these data to empirically determine the precise threshold food C:P ratio where growth rates of *Daphnia* become limited by food P content. Animal body P content, an additional indicator of consumer P nutrition, was measured across this food C:P ratio gradient and compared against animal growth responses. Using updated parameter values from these experiments, we calculated the TER_C:P_ and maximal growth rates for *Daphnia* using an established individual mass balance model^10^ and extended this model to estimate error around the TER_C:P_. Our primary objective was to estimate, empirically and theoretically, the TER_C:P_ of *Daphnia* as a means to understand the nature and frequency of P-limitation likely to be experienced by this important zooplankton consumer. Further, we compared our empirical and modeling based TER_C:P_ estimates, contrasted these estimates with previously derived literature values and discussed physiological reasons for this variation.

## Methods

### Growth experiments

Mass specific growth rate (MSGR) experiments on *D. magna* were completed over a relatively narrow range of dietary C:P ratios to empirically identify the TER_C:P_. For a food source, we grew *Scendesmus obliquus* (purchased as *S. acutus* from the Canadian Phycological Culture Centre 10) under different P supplies. Algae were grown in semi-continuous cultures at 20 °C under constant aeration and light intensity of ~150 μmol s^−1^ m^−2^. We diluted cultures daily with sterilized algal media^20^ enriched with different P concentrations. Collected algae was examined under the microscope and found primarily (>95%) unicellular regardless of the P media used to grow the cells. Using this approach, we cultured algae having a range of C:P ratios (80–350). Algae was collected each day and concentrated by centrifuging (Sorvall Legend TR) at 4066 g for 15 minutes at room temperature (~20 °C). For each experimental run, we produced algal food at one or more “elevated” ratio (C:P ratios >120) and a low C:P ratio (C:P ratio ~100). To verify algal nutrient content and density, we saved a small subsample by filtering each concentrated algal sample onto pre-weighed Whatman GF/C filters (2 ashed for CN and 2 unashed for P analysis). Filters were dried for a minimum of 2 h at 60 °C before being reweighed for mass determination and subsequent elemental analysis.

*Daphnia magna* (clone DG106) mothers were grown in jars containing 400 mL of P-free COMBO media^21^ that was changed at least three times a week and contained high quantities of high P algae (C:P ~80–100). Experiments were set up by pooling neonates under 16 h from broods 2–5 from these cultured mothers. These neonates were subsequently triple rinsed in P-free COMBO to remove all the residue food and nutrients. For growth experiments, we saved five aggregate samples of 20 neonates each that were subsequently dried to determine initial mass. The remaining neonates were placed into individual tubes containing 40 mL of P-free COMBO and fed 6 mg C L^−1^ of the prescribed diet. On day three of each experiment, we transferred all animals into new media containing a higher food concentration (8 mg C L^−1^). After 6 days of growth, animals were saved individually for MSGR (n = 8). Samples of 4 pooled individuals were also saved for body P content (n = 5, total of 20 animals) and CN analysis (n = 3, total of 12 animals). All samples were dried at 60 °C for a minimum of 72 h before being weighed. We repeated this experiment 18 times with 41 different food C:P ratios between June and August of 2015.

### Elemental analysis

We measured the P content of algae and animals after digesting filters or animal bodies with potassium persulfate using the molybdate-blue method^22^. Particulate C and N content was measured with a CN analyzer (Vario Elementar EL III).

### Statistics

MSGR was calculated by subtracting the natural log of average neonate mass of all the experiments from the natural log of the individual mass of each experimental animal and dividing by the duration of the experiment (6 days). Using least squares based optimization, a piecewise linear curve was fitted to the data of animal MSGR vs algal C:P ratios. Linear fits were made to assess relationships between *Daphnia* body C:N and C:P ratios and algal C:P ratios as piecewise regression yielded no improvement over simple linear regression for these two response variables.

### Model description

We used an individual mass balance model to estimate the TER for C:P and maximum growth rate of *D. magna*. Building off of previously published approaches^4,5,10^, we assumed balanced growth at the point of the threshold such that the rate of C increase must be matched by the rate of P increase scaled by the body C:P ratio:

1 $$\frac{{\rm{dC}}}{{\rm{dt}}}\ast \frac{1}{{\rm{C}}}=\frac{{\rm{dP}}}{{\rm{dt}}}\ast \frac{1}{{\rm{C}}}\ast \frac{{{\rm{Q}}}_{{\rm{C}}}}{{{\rm{Q}}}_{{\rm{p}}}}$$

with $\frac{{{\rm{Q}}}_{{\rm{C}}}}{{{\rm{Q}}}_{{\rm{P}}}}$ = body C:P ratio of *Daphnia*. Under steady state conditions, the incorporation of C and P into new body production will be equal and equivalent to their delivery rates from ingested food after digestion and absorption such that:

2 $$({{\rm{I}}}_{{\rm{C}}}\ast {{\rm{A}}}_{{\rm{C}}})-{{\rm{R}}}_{{\rm{C}}}={{\rm{I}}}_{{\rm{C}}}\ast {{\rm{f}}}_{{\rm{P}}/{\rm{C}}}\ast {{\rm{A}}}_{{\rm{P}}}\ast \frac{{{\rm{Q}}}_{{\rm{C}}}}{{{\rm{Q}}}_{{\rm{P}}}}$$

with I_C_ = maximum ingestion rate, A_C_ = assimilation efficiency of C, R_C_ = respiration rate, f_P/C_ = food P:C ratio, and A_P_ = assimilation efficiency of P. We rearranged Equation 2 to isolate the food C:P ratio, which is equivalent to the TER_C:P_:

3 $${\rm{f}}\frac{{\rm{C}}}{{\rm{P}}}=\frac{{{\rm{A}}}_{{\rm{p}}}}{{{\rm{A}}}_{{\rm{c}}}-\frac{{{\rm{R}}}_{{\rm{c}}}}{{{\rm{I}}}_{{\rm{c}}}}}(\frac{{\rm{Qc}}}{{\rm{Qp}}})$$

Using Equation 3, we calculated TER_C:P_ using parameter values from the literature and the current study (Table 2). To assess uncertainty in this TER, we propagated error associated with each parameter value (p_i_) to calculate the standard deviation of the TER_C:P_ (δTER) using standard error propagation methods:

4 $${\rm{\delta }}\mathrm{TER}=\sqrt{{{\sum }_{{\rm{i}}}(\frac{\partial {\rm{G}}}{\partial {{\rm{p}}}_{{\rm{i}}}}{{\rm{\Delta }}{\rm{p}}}_{{\rm{i}}})}^{2}}$$

where $\frac{\partial {\rm{G}}}{\partial {{\rm{p}}}_{{\rm{i}}}}$ indicates the partial derivative of a function with respect to the specified parameter and Δp_i_ indicates the empirical error in the parameter value.

**Table 2:** Parameters used in current TER_C:P_ model of Daphnia magna.

	Q_C_^a^	Q_P_^a^	R_C_^28^	I_C_^29^	A_C_^29,30^	A_P_^21^
	(mg C DW^−1^)	(mg P mg DW^−1^)	(day^−1^)	(day^−1^)	(unitless)	(unitless)
Mean	0.446	0.0147	0.19	0.95	0.68	0.95
st. dev.	0.018	0.0013	0.029	0.03	0.035	0.03

Shown are means and standard deviations (st. dev.) for each parameter obtained from this study or previous studies.

^a^This study.

We further examined the links between TER_C:P_ and maximum growth rates using modified Gaussian functions which take into account the functional relationship between TER_C:P_ and maximum growth rates in the individual mass balance model. This allowed us to determine the probability density in the “TER_C:P_ vs maximum growth space”. To do so, we used the following formulation:

5 $${\rm{\psi }}({\rm{m}},{\rm{TER}})=(\frac{1}{{{\rm{\sigma }}}_{{\rm{m}}}\sqrt{2{\rm{\pi }}}}{{\rm{e}}}^{-\frac{{({\rm{m}}-{{\rm{m}}}_{{\rm{o}}})}^{2}}{2{{\rm{\sigma }}}_{{\rm{m}}}^{2}}})(\frac{1}{{{\rm{\sigma }}}_{{\rm{TER}}}\sqrt{2{\rm{\pi }}}}{{\rm{e}}}^{-\frac{{({\rm{TER}}-{\rm{f}}({\rm{m}}))}^{2}}{2{{\rm{\sigma }}}_{{\rm{TER}}}^{2}}})$$

where m is the maximum growth rate being considered and TER is the threshold being considered. The f(m) term is the model functional relationship between the threshold C:P and m given by:

6 $${\rm{f}}({\rm{m}})=\frac{{{\rm{I}}}_{{\rm{C}}}{{\rm{A}}}_{{\rm{p}}}({{\rm{Q}}}_{{\rm{C}}}/{{\rm{Q}}}_{{\rm{P}}})}{{\rm{m}}}$$

with the m_o_ term indicating the calculated maximum growth rate according to the empirical growth parameters as follows:

7 $${{\rm{m}}}_{{\rm{o}}}=\frac{{{\rm{I}}}_{{\rm{C}}}{{\rm{A}}}_{{\rm{P}}}({{\rm{Q}}}_{{\rm{C}}}/{{\rm{Q}}}_{{\rm{P}}})}{{{\rm{f}}}_{{\rm{C}}:{\rm{P}}}}$$

The σ_TER_ value is the standard deviation for TER and σ_m_ is standard deviation for maximum growth. These were propagated from the empirical parameters according to equation 4. Combining all of this into a normalized 2D Guassian results in equation 5. Along with this, we determined a confidence region for the relationship by tracing the specific probability density at which 95% of the values are expected to be contained in a standard 2D Guassian distribution. As we used a normalized Equation 5, the integral of the probability density over the region is equal to 0.95, meaning with the parameters and parameter errors given, there is a 95% chance that the region contains the true TER and maximum growth combination.

### Data accessibility

The datasets generated during and/or analysed during the current study are available from the corresponding author on reasonable request.

## Results

### Growth and body elemental composition responses

**Figure 1:**
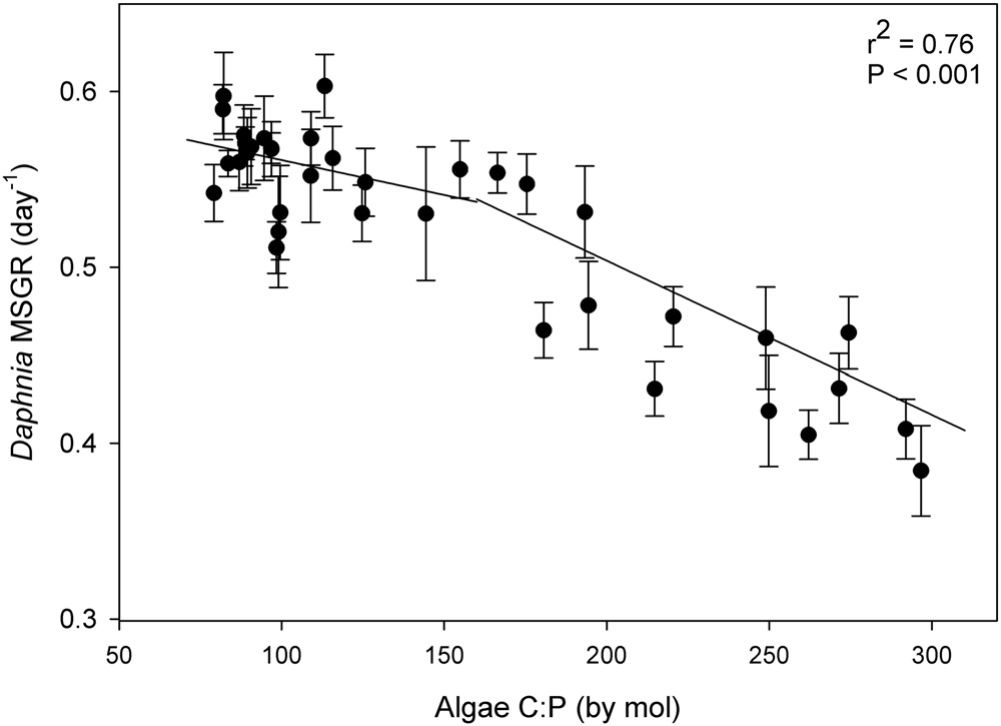
Mass-specific growth rates (MSGR) of *Daphnia magna* as they relate to algal C:P ratios. Shown are means and standard deviation of each run (n = 8). Relationships are shown through partial least squares regression.

*Daphnia* growth rate decreased with higher food C:P ratios (Fig. 1). Using piecewise linear regression, we found a break-point in growth rates at a food C:P ratio of 155 ± 14. Above this break-point, daphnid growth rates decreased more steeply with increasing food C:P ratios. Animals reduced their growth rate by 25–30% at food C:P ratios of 200–300, compared to the growth rates for food C:P <130 (Fig. 1). At food C:P ratios lower than the breakpoint ratio of 155, daphnid growth rates still decreased with increasing C:P, however, this was at a significantly lower rate (Fig. 1).

**Figure 2:**
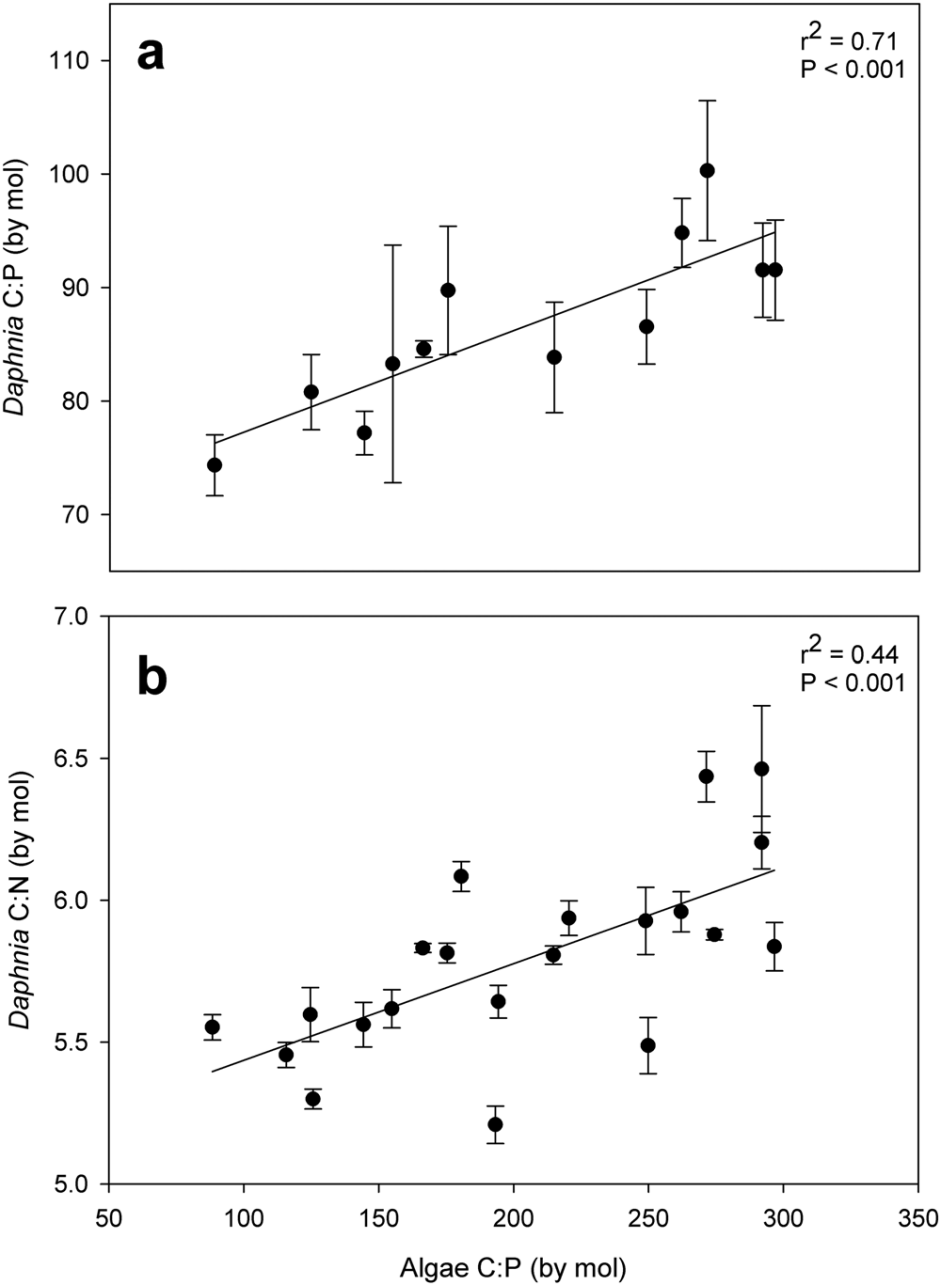
*Daphnia magna* body stoichiometry across algal C:P gradients. Each point represents the mean and standard deviation of body (**a**) C:P and (**b**) C:N measurements (n = 4) for each separate food C:P ratio. Relationships are determined by ordinary least squares regressions.

*Daphnia* body C:N and C:P ratios both increased with food C:P ratios across the 100 to 300 range (Fig. 2). We found no apparent break-point that would be consistent with a TER at any point along the food C:P ratio gradient. Both ratios were linearly related to food C:P ratios and were not accompanied by non-normal residual error. Daphnid body C:P ratios displayed a steeper slope with more variation explained against food C:P ratios compared to body C:N ratios (Fig. 2).

### Modelled threshold elemental ratios and growth rates

**Figure 3:**
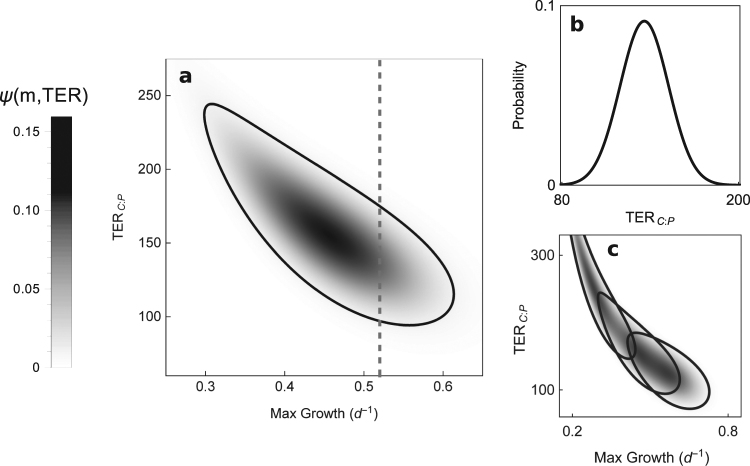
Modelled threshold elemental ratios of carbon and phosphorus in *Daphnia*. (**a**) Probability vs TER_C:P_ and maximum growth rates estimated by individual mass-balance model using parameter values and error found in Table 2. The z scale (amount of shading) indicates how probable it is that with the specified parameters, *Daphnia* will experience the TER_C:P_ and maximum growth combination indicated by the axis values. The solid line surrounding the region of high probability represents the 95% confidence region (**b**) Direct slice of plot a (indicated by dotted line) through a maximum growth rate of 0.52 as indicated by the dotted line in a. (**c**) TER_C:P_ versus maximum growth rates for calculated with different assimilation efficiencies. 95% confidence region for each are circled A_c_ = 0.5 (left), A_c_ = 0.68 (middle) and A_c_ = 0.8 (right).

The TER_C:P_ calculated using Equation 3 and its standard deviation with the error propagation approach (Equation 4) was found to be 155 ± 16. As dictated in Equation 7, increasing maximum growth rate in our model was accompanied by lower TER_C:P_, which is apparent in our probabilistic density analysis (Fig. 3). In this analysis, the TER_C:P_ associated with our observed maximum growth rates (~0.52 day^−1^) had a value of ~140. While our analysis also found that higher TER_C:P_ are possible given the parameter values and their error used in our model, these parameter value sets were associated with relatively slow maximum growth rates (<0.4 day^−1^) of *Daphnia* that are well below those measured here and previously reported in the literature. We calculated relatively high TER_C:P_ (>200) when using lower A_C_ values (0.50) but found low maximum growth rates (<0.3 day^−1^) in these model runs (Fig. 3).

## Discussion

We found that the TER_C:P_ of *Daphnia magna* to be ~155 based on both growth measurements and with refined parameter values in an individual mass balance model^10^. Our empirical estimate of *Daphnia*’s TER_C:P_ was generated using a fine-scale food quality gradient that allowed us to carefully examine animal growth responses to poor food quality near the TER. Our modelled TER_C:P_ estimate included updated parameter values and propagated error terms, which provided an estimate of the threshold with a known level of certainty. Our current estimates are similar to TER_C:P_ estimates produced by similar modelling efforts (Table 1) but are substantially lower than TER_C:P_ estimates from two other studies (385, Urabe and Watanabe^11^; 300, Brett *et al*.^20^). A low TER_C:P_ of ~155, as found here, indicates that *D. magna* may be especially prone to experience P-limitation in its food^10^ and that food with even moderately low food C:P ratios (200) will have negative effects on its growth and reproduction.

Our results demonstrate slower growth in *Daphnia* consuming slightly elevated food C:P ratios (~200) compared to those animals feeding on food C:P ratios at or below the TER_C:P_ (~150). This reduction in growth rates and the changes observed in animal body P content with food C:P ratios above the threshold is consistent with expectations and previous observations of P-limited growth in animals^23,24^ at higher food C:P ratios. At or near the calculated TER_C:P_ , our growth experiments found no evidence of an obvious stoichiometric ‘knife-edge’ as even below our regression break-point, *Daphnia* growth rates continued to increase with decreasing food C:P ratios. As we did not reduce food C:P ratios below 80, we cannot rule out possible declines in growth rates with lower food C:P ratios (<60−70). It may be that the stoichiometric knife-edge is not a sharp transition point and instead manifests itself as a stoichiometric ‘hill-top’. This lack of abrupt changes in growth above and below the threshold may be because of physiological acclimation to nutrient-deficient food, which allows an organism to sustain growth even when presented with slightly imbalanced food^1^.

We used a mass balance formulation to calculate a theoretical estimate of the TER_C:P_ for *Daphnia*. This model formulation has been previously used with *Daphnia* in several earlier studies to estimate TER_C:P_ of 200 or below (Table 1). Our current model formulation, simplistic compared to some of these studies (e.g.,^25^), is derived from Frost *et al*.^10^ under the assumptions of high food abundance and no possible limitation by a third element. The current application of this model of *Daphnia* TER_C:P_ differed from past studies by incorporating uncertainty in measured parameter values and yielded a calculated TER_C:P_ with a 95% confidence interval between 123–187. We further constrained the probable range of TER_C:P_ range by examining the relationship between the modelled TER_C:P_ and the maximum MSGR predicted by the model. Given that measured maximum growth rates are typically ~0.50 day^−1^, we excluded TER_C:P_ estimates that were associated with much faster or slower growth. The TER_C:P_ estimate (~140) associated with this growth rate is nearly the same as our experimentally determined point and to the average modelled TER_C:P_ as described above.

We found that an elevated TER_C:P_ (e.g., >200) could be calculated using this TER model formulation but these higher threshold ratios were associated with unrealistic sets of parameter values. Combinations of parameters that yielded elevated TER_C:P_ for *Daphnia* were generally inconsistent with parameter values reported in the literature and when combined to calculate growth yielded unrealistically low maximum specific growth rates. For example, lower ingestion rates, higher respiration rates, and low assimilation efficiency of C all reduce the net intake and incorporation of C by *Daphnia* and, in the TER model, parameter values associated with these physiological states yielded higher estimates of the TER_C:P_. Such changes in animal acquisition of C would severely constrain maximal animal growth rates below that typically measured for laboratory animals and are inconsistent with observations of physiological processes in well-nourished, fast growing *Daphnia*. This theoretical coupling of TER and growth rates thus provides a quantitative approach to assessing whether parameters used to calculate thresholds are realistic and to verify that TER estimates for a given species are consistent with the known biology of the species under consideration. These connections also indicate that species-specific differences in nutrient acquisition, while largely beyond the scope of this study, should be examined for their connections to maximum attainable growth rates.

We estimated the TER_C:P_ using the mass balance formulations, which have been extensively analyzed by previous studies^10,25,26^. The past analyses have indicated that caution is warranted when using this approach due, in part, to parameters in the model being themselves sensitive to food quality^10,25^. For example, sustained P-limitation can dramatically reduce carbon use efficiency^15,23^, which, if represented in a TER model, yields a relatively high TER_C:P_ and the contradictory conclusion that P-limitation is unlikely for this taxon^14^. A previous examination of this problem recommended using parameter values that were based on first principles and well-grounded in the biology of the organism^25^. Another solution is to only use parameter values estimated on animals growing maximally under conditions of high quantities of good food quality, which thus match the conditions assumed to be operating at the TER. Estimates of TER that incorporate parameters derived from animals growing under food or nutrient-limiting conditions should thus be considered unreliable and may account for some of the previously reported differences in thresholds estimated for *Daphnia* and other taxa.

Modelled TER estimates are also sensitive to physiological parameters that are known to vary with organismal traits, life-history, trophic position, environmental conditions, and other forms of physiological stress^6,10,27^. Against this, it is valid to question whether a TER_C:P_ determined under single set of laboratory conditions can be applied to a broader context. Laboratory derived TER_C:P_ estimates provide baseline information on consumer nutritional requirements, in the absence of other environmental factors, that can be used understand the nutritional circumstances under which an organism would be nutrient-limited in the field. One could imagine incorporating additional sources of variation such as multiple limiting elements, physico-chemical conditions (e.g., temperature), and other food traits (e.g, digestibility or lipid composition) to yield multidimensional TER estimates^28^. Future work should examine the sensitivity of TER to these other conditions and couple this information with data from emerging nutritional indicators^2^ to better define conditions, dietary or otherwise, that separate multiple forms of nutritional limitation on ecologically important consumer taxa. In addition, the applicability of growth-related TER estimates to other response variables such as age at first reproduction and reproductive effort would be worthwhile given the possibly different nutritional demands of these alternative life-history traits^3^. Nonetheless, while physiological parameters (e.g., feeding rates and body stoichiometry) can vary among species and TER estimates should be viewed as species-specific, mass balance constraints underlying the TER formulation place limits on TER and growth rates that are unavoidable regardless of taxonomic identity.

This study aimed to better understand the TER_C:P_ of *Daphnia magna* by combining growth experiments and metabolic models. Our work found a relatively high demand for P for *Daphnia* that makes it especially susceptible to dietary P- limitation. While our model results demonstrate physiological adjustments could potentially modulate these P demands and reduce the likelihood of P-limited growth, this type of acclimation would necessarily be accompanied by dramatically slower growth. Consequently, there is a link between increasing the acquisition of the limiting element relative to other elements and the ability of an animal to grow maximally. This trade-off appears to complement other molecular complexities^29^ that dictate strong connections between fast growth and elevated P demands in organisms. Future work should try to couple these alternative views of animal growth physiology to better understand how material constraints affect growth processes and nutrient dynamics as advocated by the theory of ecological stoichiometry^3^.
